# Overexpression of a C_4_-dicarboxylate transporter is the key for rerouting citric acid to C_4_-dicarboxylic acid production in *Aspergillus carbonarius*

**DOI:** 10.1186/s12934-017-0660-6

**Published:** 2017-03-14

**Authors:** Lei Yang, Eleni Christakou, Jesper Vang, Mette Lübeck, Peter Stephensen Lübeck

**Affiliations:** 1 Section for Sustainable Biotechnology, Department of Chemistry and Bioscience, Aalborg University Copenhagen, A. C. Meyers Vænge 15, 2450 Copenhagen SV, Denmark; 2 Section for Biotechnology, Aalborg University Copenhagen, Fredrik Bajers Vej 7H, 9220 Aalborg, Denmark

**Keywords:** *Aspergillus carbonarius*, Citric acid, C_4_-dicarboxylate transporter, Lignocellulosic biomass, Malic acid, Metabolic engineering, Succinic acid

## Abstract

**Background:**

C_4_-dicarboxylic acids, including malic acid, fumaric acid and succinic acid, are valuable organic acids that can be produced and secreted by a number of microorganisms. Previous studies on organic acid production by *Aspergillus carbonarius,* which is capable of producing high amounts of citric acid from varieties carbon sources, have revealed its potential as a fungal cell factory. Earlier attempts to reroute citric acid production into C_4_-dicarboxylic acids have been with limited success.

**Results:**

In this study, a glucose oxidase deficient strain of *A. carbonarius* was used as the parental strain to overexpress a native C_4_-dicarboxylate transporter and the gene *frd* encoding fumarate reductase from *Trypanosoma brucei* individually and in combination. Impacts of the introduced genetic modifications on organic acid production were investigated in a defined medium and in a hydrolysate of wheat straw containing high concentrations of glucose and xylose. In the defined medium, overexpression of the C_4_-dicarboxylate transporter alone and in combination with the *frd* gene significantly increased the production of C_4_-dicarboxylic acids and reduced the accumulation of citric acid, whereas expression of the *frd* gene alone did not result in any significant change of organic acid production profile. In the wheat straw hydrolysate after 9 days of cultivation, similar results were obtained as in the defined medium. High amounts of malic acid and succinic acid were produced by the same strains.

**Conclusions:**

This study demonstrates that the key to change the citric acid production into production of C_4_-dicarboxylic acids in *A. carbonarius* is the C_4_-dicarboxylate transporter. Furthermore it shows that the C_4_-dicarboxylic acid production by *A. carbonarius* can be further increased via metabolic engineering and also shows the potential of *A. carbonarius* to utilize lignocellulosic biomass as substrates for C_4_-dicarboxylic acid production.

## Background

C_4_-dicarboxylic acids, including malic acid, fumaric acid and succinic acid, are amongst top value added chemicals with their large and growing markets due to wide spectra of applications [1]. In addition to their traditional uses such as food additives, chelators and acidulants, C_4_-dicarboxylic acids have been for the past decades extensively exploited to be key building blocks for deriving varieties of commodity and specialty chemicals [2]. To reduce the dependence of the global economy on petroleum industry, bio-refinery of renewable biomass is considered to be an alternative approach to support industrial manufacture [3]. The fact that C_4_-dicarboxylic acids are present as key intermediates in primary metabolism of living cells indicates the potential of using microbial systems to produce them from fermentable sugars derived from renewable biomass and the feasibilities of improving the production strains via metabolic engineering [4–6]. In recent years, bio-based production of C_4_-dicarboxylic acids has received increasing research attention and achieved an important status in bio-economy. So far, bio-based succinic acid production has succeeded with a number of commercialized processes using bacterial strains (*Escherichia coli* and *Actinobacillus succinogenes*) and yeast strains (*Saccharomyces cerevisiae*) [7], and biotechnological processes for malic acid and fumaric acid are under research development [8, 9]. The bottlenecks in the current biotechnologies for production of C_4_-dicarboxylic acids are relatively low productivity in production processes and high production cost due to the choice of substrates (glucose) and downstream product purification [10]. Although the research effort to address those technical constraints now mainly focus on the industrial candidate strains, exploiting new cell factories with their special genetic and physiological traits may open the window of opportunity for future technical breakthrough in the bio-based production of C_4_-dicarboxylic acids.

Application of fungal technology in industrial production of organic acids has been demonstrated with several *Aspergillus* species, such as citric acid production by *Aspergillus niger* and itaconic acid production by *Aspergillus terreus* [11–13]. *Aspergillus carbonarius*, a member of black aspergilli, possesses several valuable virtues to be a competent cell factory for organic acid production. It can produce different types of organic acids (citric acid, gluconic acid and malic acid) from varieties of substrates ranging from mono-sugars to polysaccharides such as glucose, xylose, cellulose and starch, and tolerate stress conditions, especially low pH, during organic acid production [14, 15]. Organic acid profiling of *A. carbonarius* has shown its capability of producing high amounts of citric acid and gluconic acid under different pH conditions [16]. To further strengthen its abilities for organic acid production, a series of genetic modifications has been introduced targeted to the primary metabolic pathways and the regulatory system in *A. carbonarius* [14, 17, 18]. However, significant impacts have only been obtained on the production of citric acid rather than other organic acids e.g. C_4_-dicarboxylic acids. Deletion of glucose oxidase that converts glucose to gluconic acid in pH buffered conditions that are suitable for C_4_-dicarboxylic acid production, completely eliminated accumulation of gluconic acid and increased citric acid production, but only improved malic acid production at a very limited level [18]. Overexpression of enzymes carrying out the carboxylation of phosphoenolpyruvate in *A. carbonarius* supposed to increase the carbon flux towards the rTCA branch from which C_4_-dicarboxylic acids are produced as key intermediates [19], gave no significant impact on the production of C_4_-dicarboxylic acids, and the increased carbon flux seemed to flow towards citric acid production [16]. This phenomenon was also observed in another well-known citric acid producing species, *A. niger*, when three genes involved in the rTCA branch were overexpressed [20]. In addition to central carbon metabolic pathways, export of C_4_-dicarboxylic acids is an essential step to consider in metabolic engineering of microbial strains for production of C_4_-dicarboxylic acids. In *Aspergillus oryzae* and *S. cerevisiae*, synergistic impacts on malic acid production were obtained when C_4_-dicarboxylate transporters were overexpressed in combination with other genetic modifications [9, 21]. In *A. carbonarius*, there is not yet any report regarding the C_4_-dicarboxylate transporter.

**Fig. 1:**
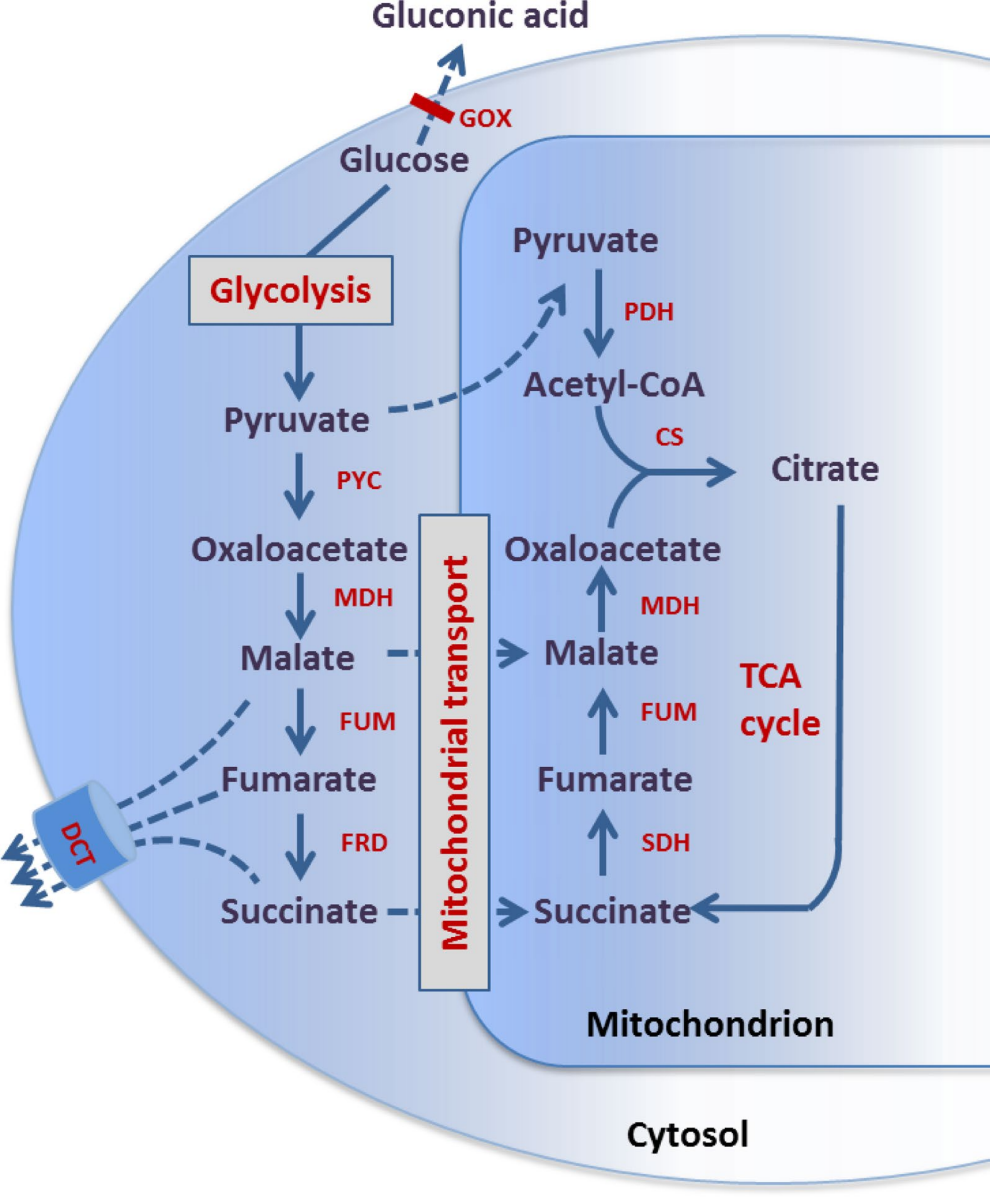
Proposed metabolic pathway for C_4_-dicarboxylic acid production by *A. carbonarius* PYC, pyruvate carboxylase; MDH, malate dehydrogenase; FUM, fumarase; FRD, fumarate reductase; DCT, C_4_-dicarboxylate transporter; PDH, pyruvate dehydrogenase; CS, citrate synthase; SDH, succinate dehydrogenase and TCA cycle, tricarboxylic acid cycle

In this study, we identified a gene *dct* encoding a putative C_4_-dicarboxylate transporter (DCT) from the genome of *A. carbonarius* and overexpressed it in a glucose oxidase deficient strain to examine its effect on C_4_-dicarboxylic acid production. The *∆gox* strain is used as a parental strain as it provides an ideal platform to evaluate the impact of introduced genetic modifications in glucose containing media under pH buffered conditions without the interference of extracellular conversion of glucose to gluconic acid [18]. Furthermore, we expressed the *frd* gene encoding a NADH dependent fumarate reductase from *Trypanosoma brucei* in combination with the *dct* gene to increase succinic acid production (Fig. 1).

## Methods

### Strains and cultivation conditions

A glucose oxidase deficient *∆gox* strain, which is not able to produce gluconic acid [18], was selected as the parental strain to construct the derived strains in this study. *A. carbonarius* wild type ITEM5010 was used as a donor strain for obtaining the *dct* gene. The cultivation of the strains for spore production was carried out in potato dextrose agar medium at 30 °C for 4–6 days. For purification of total RNA, strains were inoculated into yeast extract peptone dextrose (YEPD) medium and incubated stationary at 30 °C for 2 days.

### Identification of a C_4_-dicarboxylate transporter in *A. carbonarius*

The amino acid sequences of the C_4_-dicarboxylate transporter (C4T318) in *A. oryzae* and the malic acid transporter (MAE1) in *Schizosaccharomyces pombe* (accession no. BAE58879.1 and NP594777.1) were selected to identify an orthologous gene in *A. carbonarius.* The amino acid sequence with high identity was identified as the putative C_4_-dicarboxylate transporter and the encoding gene was termed *dct* (accession no. KY178298) in this study.

### Plasmid construction and fungal transformation

**Fig. 2:**
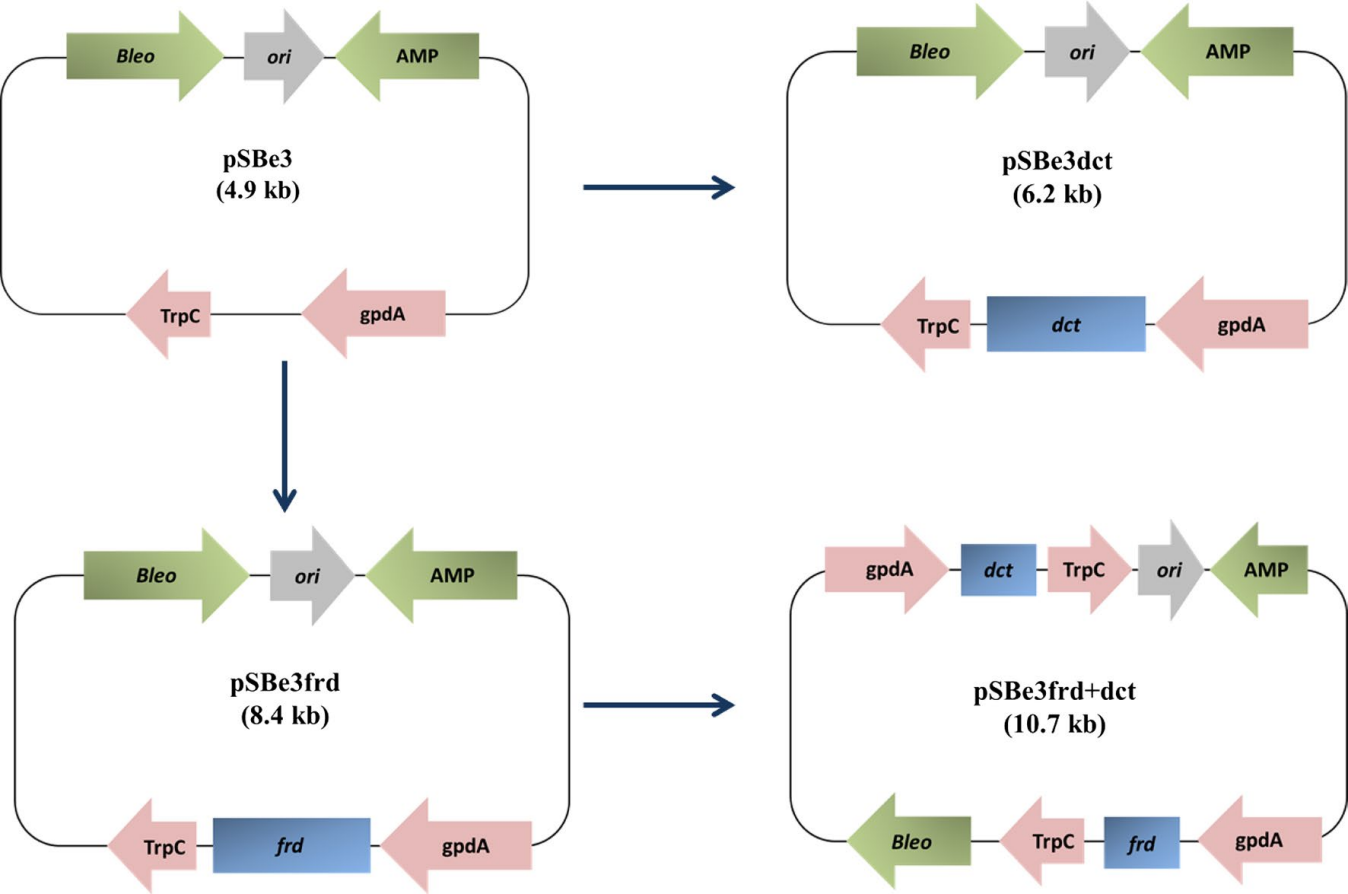
Plasmids used in this study *gpdA,* constitutive promoter; *TrpC*, terminator, *Bleo,* phleomycin resistance gene; *AMP* ampicillin resistance gene; *frd,* fumarate reductase encoding gene; *dct,* dicarboxylate transporter encoding gene; *ori*, origin of replication in the plasmid

The *dct* gene encoding the putative C_4_-dicarboxylate transporter was amplified with primers DctFw and DctRv from the cDNA that was synthesized from total RNA of the wild type as previously described [16]. The *frd* gene encoding a NADH dependent fumarate reductase in *Trypanosoma brucei* was codon optimized, synthesized and cloned as previously described [22]. Both genes were inserted individually via Simple USER cloning [23] into a fungal expression cassette consisting of a constitutive promoter gpdA and a terminator TrpC in plasmid pSBe3 that carries the phleomycin resistance gene *bleo* (Fig. 2). For co-transformation, the *dct* gene flanked with the gpdA promoter and TrpC terminator regions were amplified with primers GpdFw and TrpRv from plasmid pSBe3dct and inserted into plasmid pSBe3frd (Table 1 and Fig. 2) to construct pSBe3frd-dct. All resulting plasmids were verified by DNA sequencing (StarSEQ^®^).

**Table 1:** Primers used in this study

Name	Sequence (5′ → 3′)	Annotation
DctFw	AGAGCGAUATGCATGTCCACGACACC	SimpleUSER cloning of *dct* gene
DctRv	TCTGCGAUTCATTCAGACACATCCTCGTC	SimpleUSER cloning of *dct* gene
FrdFw	AGAGCGAUATGGTTGATGGTCGGTCGT	SimpleUSER cloning of *frd* gene
FrdRv	TCTGCGAUTTAGCTACCCGACGGTTCAGTT	SimpleUSER cloning of *frd* gene
GpdFw	GGCATTAAUTCGTGGACCTAGCTGATTCTG	PCR amplification of expression cassette
TrpRv	GGTCTTAAUTCGAGTGGAGATGTGGAGTG	PCR amplification of expression cassette
qDctFw	TTCCTTCCACCTCAACTGGT	qPCR *dct* gene
qDctRv	GCTGAGCAGGACAAAGATGA	qPCR *dct* gene
qActinFw	AGAGCGGTGGTATCCATGAG	qPCR beta-actin gene
qActinRv	TGGAAGAGGGAGCAAGAGCG	qPCR beta-actin gene

Protoplast transformation was carried out with the above mentioned plasmids as previously described [18]. Minimal medium with agar (MMA) consisted of glucose, 10 g/L; sorbitol, 182 g/L; NaNO_3_, 6 g/L; KCl, 0.5 g/L; MgSO_4_·7H_2_O, 0.5 g/L; KH_2_PO_4_, 1.5 g/L; ZnSO_4_, 0.005 g/L; FeSO_4_·7H_2_O, 0.003 g/L; CuSO_4_, 0.001 g/L; MnCl_2_, 0.002 g/L; biotin, 0.001 g/L; thiamine, 0.001 g/L; riboflavin, 0.001 g/L; para-aminobenzoic acid, 0.001 g/L; agar, 18 g/L and 100 µg/mL phleomycin. To stabilize the derived transformants from protoplast transformation, the spores from transformants were streaked out on potato dextrose agar (PDA) plate and pieces of agar with single colonies were transferred back to MMA. This stabilization procedure was repeated three times before the obtained transformants were verified for the transformed genes by PCR.

### cDNA synthesis and transcription analysis

For cDNA synthesis, total RNA was extracted from fresh fungal mycelia with a total RNA isolation kit (A&A Biotech^®^) and treated with DNaseI (Thermo Scientific^®^) according to the manufacturer’s manuals. The cDNA was synthesized from the treated RNA samples with a reverse transcription kit (Bio-Rad^®^) as previously described [16]. For transcription analysis of *dct* gene, quantitative real-time PCR (qPCR) was set up by mixing Ultra-Fast SYBR green qPCR mix, the synthesized cDNA and corresponding primers according to the manufacturer’s manual. The qPCR reaction was carried out in a Rotor-Gene 6000 RT-PCR machine using the following program: initial incubation at 95 °C for 5 min; 40 cycles of at 95 °C for 20 s. and 60 °C for 35 s. The threshold cycle (Ct), baseline and efficiency of amplification were determined in RG6000 application software. The relative expression level of the *dct* gene was calculated by normalizing the gene expression level of the *dct* gene to the reference gene beta-actin based on Ct values obtained from biological triplicates, and changes of the *dct* gene expression level between the selected transformants and parental strain were calculated using the 2^−ΔΔCt^ method [24].

### Enzyme assay of fumarate reductase

The expression of fumarate reductase in the selected transformants was verified with measurement of fumarate reductase activity. The *frd* overexpression strain and the parental strain were cultivated in the YPED medium for 2 days and the fresh grown mycelia was used for preparation of cell extract. The cell extract was prepared as previously described [22], and the cytosolic faction was used immediately for measurement of enzyme activity. Fumarate reductase was assayed spectrophotometrically in 1.5 mL cuvettes (1.0 cm light path) at 30 °C. The assay mixture was composed of 50 mM phosphate buffer (pH 6.5), 20 mM fumarate, 0.2 mM NADH and the enzyme activities were determined by monitoring the absorbance due to the oxidation of NADH at 340 nm for 10 min [25]. One unit fumarate reductase activity was defined as the oxidation of 1 μmol NADH based on the amount of protein per minute at 30 °C and pH 6.5

### Organic acid production

All the strains in this study were firstly cultivated in a pre-culture medium containing 3.6 g/L yeast extract and 10 g/L peptone. The freshly harvested spores were inoculated in pre-culture medium at final concentration of 10^5^/mL. The pre-cultivation was carried out in 50 mL falcon tubes containing 10 mL pre-culture medium at 30 °C for 2 days with agitation speed of 180 rpm. After pre-cultivation, the pre-culture was filtered with Mira-cloth and the pre-grown fungal cells were transferred into the production medium. The cultivation was carried out in 100 mL Erlenmeyer flasks containing 20 mL acid production (AP) medium at 30 °C with agitation speed of 180 rpm. The defined medium consisted of glucose, 80 g/L; NH_4_NO_3_, 1.5 g/L; KH_2_PO_4_, 0.15 g/L; MgSO_4_·7H_2_O, 0.8 g/L; CaCl_2_·2H_2_O, 0.2 g/L; NaCl, 0.15 g/L; ZnSO_4_, 0.0015 g/L; FeSO_4_·7H_2_O, 0.03 g/L; biotin, 1 × 10^−5^ g/L and CaCO_3_, 80 g/L [26]. The liquid fraction of the wheat straw hydrolysate was prepared as previously described and supplemented with the same amounts of nutrients (except glucose and xylose) and calcium carbonate as mentioned in the defined medium [15]. The pH of hydrolysate was adjusted to 6.5 using 10 M NaOH followed by sterilization with 0.2 µm sterile filter (Nalgene^®^). The initial concentration of glucose and xylose in the sterilized hydrolysate were 66 and 55 g/L respectively.

### Analysis of sugars and organic acids

All samples were acidified and filtered before HPLC analysis. Acidification of samples was achieved by adding 50 µL 50% sulfuric acid into 1 mL samples. The acidified samples were then incubated at 80 °C for 15 min and centrifuged at 14,000 rpm for 1 min. The supernatant was filtered with 0.45 µM filter before sampling for HPLC analysis. Analysis of sugars and organic acids were carried out with an Aminex 87H column (Biorad^®^) at 60 °C by using a HPLC mobile phase (5 mM H_2_SO_4_) at a flow rate of 0.6 mL/min. Malic acid in the samples was analyzed with a l-malate assay kit (Megazyme^®^).

### Fungal biomass measurement

For measurement of fungal biomass, 30 mL culture was acidified with 1 N·HCl to dissolve insoluble calcium carbonate and filtered with filter paper followed by thoroughly washing with distilled water. The washed fungal biomass was dried on filter paper at 100 °C for 48 h before weighing. All the filter papers were dried at 100 °C for 24 h before use.

## Results

### Identification of C_4_-dicarboxylate transporter and fungal transformation

**Fig. 3:**
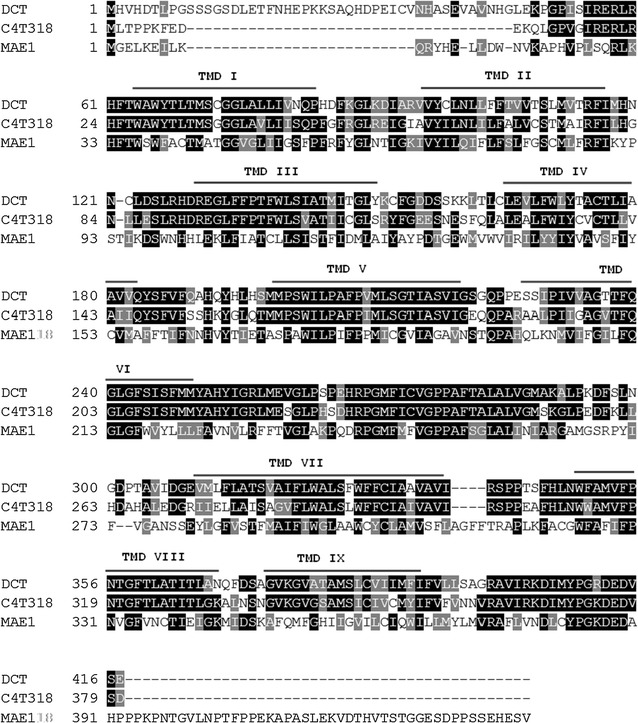
Amino acid sequence alignment of C4-dicarboxylate transporters in *A. carboanrius* (DCT), *A. oryzae* (C4T318) and *S. pombe* (MAE1) (Please note that the positions of transmembrane domains (TMD) were predicted and annotated based on the amino acid sequence of C4T318 from *A. oryzae)*

The *dct* gene was identified using two amino acid sequences of reported C_4_-dicarboxylate transporters in fungi. As seen in Fig. 3, the amino acid sequence of the C_4_-dicarboxylate transporter in *A. carbonarius* shows high identity (68%) to the reported C_4_-dicarboxylate transporter from *A. oryzae*, especially in the predicted transmembrane domains whereas the MAE1 sequence from *S. pompe* has much lower (35%) identity to the DCT in *A. carbonarius*.

Protoplast transformation of the parental strain (*∆gox)* with three plasmids (pSBe3frd, pSBe3dct and pSBe3frd + dct) resulted in 8 *frd* transformants, 12 *dct* transformants and 6 *frd*-*dct* transformants, respectively. The integration of the transformed expression cassettes containing target genes was verified with PCR amplification of the intact expression cassette. The transformants that produced the highest titers of C_4_-dicarboxylic acids in the first screening were selected for the following study and comparison.

### Overexpression of the C_4_-dicarboxylate transporter and expression of the fumarate reductase

Overexpression of the *dct* gene was verified by comparing relative expression level of the *dct* gene in the *dct* and *frd*-*dct* strains with the parental strain. The relative expression level of the *dct* gene in the *dct* and *frd*-*dct* strains increased 33- and 39-folds respectively compared with the parental strain (Table 2). Heterologous expression of fumarate reductase was confirmed in the *frd* strain and the *frd*-*dct* strain by measuring the activities of fumarate reductase. Significant activities of fumarate reductase were detected in both of the *frd* and *frd*-*dct* strains (0.013 and 0.018 U/mg) but there was no detectable activity in the parental strain (Table 3).

**Table 2:** Relative expression level of the *dct* gene in overexpressing strains

	Fold change
Parental strain (*∆gox)*	Set to 1
*dct* strain	33
*frd*-*dct* strain	39

**Table 3:** The specific activity of fumarate reductase in the parental strain *∆gox* and the FRD overexpressing strains

	Fumarate reductase
Parental strain (*∆gox)*	n.d
*frd* strain	0.013
*frd*-*dct* strain	0.018

The enzyme activity (U/mg protein) was measured in the cells after 40 h of incubation in the YPD medium

### Impacts of overexpressing the C_4_-dicarboxylate transporter and fumarate reductase on organic acid production

**Fig. 4:**
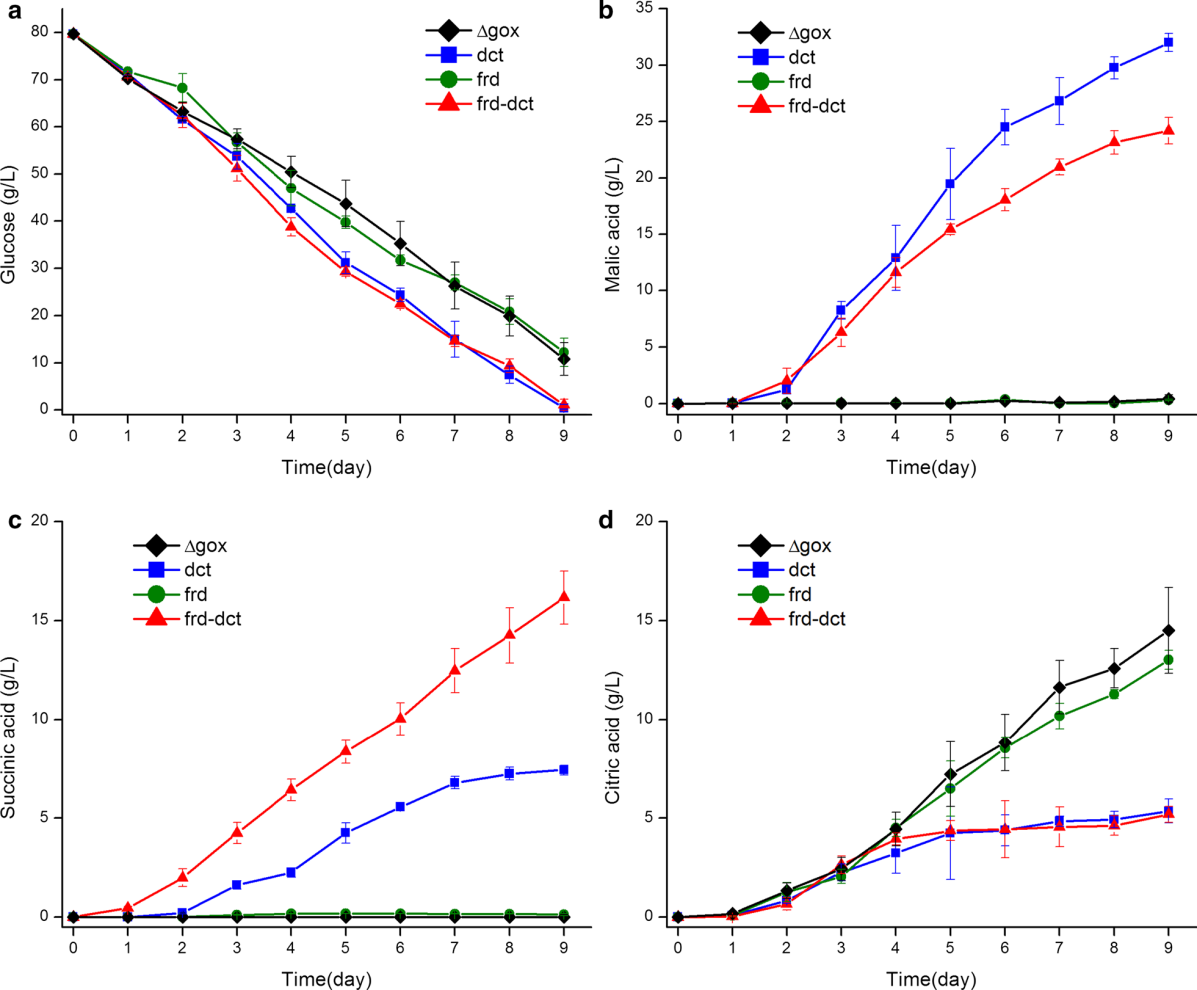
Glucose consumption and production of major organic acids in the defined medium **a** Glucose consumption (g/L); **b** production of malic acid (g/L); **c** production of succinic acid (g/L); **d** production of citric acid(g/L)

Comparison of organic acid production by the selected derived strains and the parental strain were at first made in a defined medium containing 80 g/L glucose under pH buffered condition. Overexpression of the *dct* gene substantially increased C_4_-dicarboxylic acid production in the *dct* and *frd*-*dct* strains (Fig. 4b, c; Table 4). From day 2, the two strains (*dct* and *frd*-*dct*) began to secrete malic acid and succinic acid simultaneously with production of citric acid, whereas the *frd* strain and the parental strain only produced citric acid (Fig. 4b–d). However, it seems that increased production of malic acid and succinic acid in the *dct* and *frd*-*dct* strains did not have any significant impact on citric acid production in the early phase of organic acid production. After day 4 a deceleration of citric acid production by these two strains was observed whereas the parental strain and the *frd* strain continued producing citric acid. The *dct* and the *frd*-*dct* strains continued producing malic acid and succinic acid as the glucose was consumed during the cultivation. In addition, low amounts of fumaric acid were detected in the *dct* and the *frd*-*dct* strains after 9 days (Table 4). Expression of fumarate reductase in the *frd* strain did not lead to an overproduction of succinic acid (Fig. 4c), only a slight elevation was found compared to the parental strain. Citric acid was still produced in the *frd* strain as the only major organic acid at high quantity similar to the organic acid profile obtained in the parental strain (Fig. 4b–d). When the C_4_-dicarboxylate transporter was overexpressed in combination with the fumarate reductase in the *frd*-*dct* strain, succinic acid production increased dramatically. After 9 days, the *frd*-*dct* strain produced 16 g/L succinic acid which was significantly higher than titers obtained from the *dct* strain (7.4 g/L) and the *frd* strain (0.13 g/L) (Table 4). Production of malic acid and fumaric acid was lower in the *frd*-*dct* strain compared with the *dct* strain.

**Table 4:** Comparison of sugar consumption, production of organic acids and fungal biomass in the defined medium after 9 days

Strain	Glucose consumption	Concentration (g/L)				Yield (mg/g glucose)				Fungal biomass
		Malic acid	Fumaric acid	Succinic acid	Citric acid	Malic acid	Fumaric acid	Succinic acid	Citric acid	
*∆gox*	69	0.4	n.d	n.d	14	5.8	n.d	n.d	203	461
*dct*	79	32	0.96	7.4	5.3	404	12	93	66	352
*frd*	67	0.31	n.d	0.13	13	4.6	n.d	1.9	192	459
*frd*-*dct*	79	24	0.8	16	5.2	307	10	205	66	364

C_4_-dicarboxylic acid production from wheat straw hydrolysate

From day 4 efficient production of C_4_-dicarboxylic acids also increased glucose consumption by the *dct* and *frd*-*dct* strains compared with the parental strain and the *frd* strain (Fig. 4a). After 9 days, glucose was almost depleted by the *dct* and *frd*-*dct* strain but there was still over 10 g/L glucose left in the *frd* strain and the parental strain. There were also significant differences in fungal biomass among the strains. The *dct* and *frd*-*dct* strains produced lower fungal biomass (352 mg/g glucose and 364 mg/g glucose) than parental strain and *frd* strain (461 mg/g glucose and 459 mg/g glucose) after 9 days (Table 4).

**Fig. 5:**
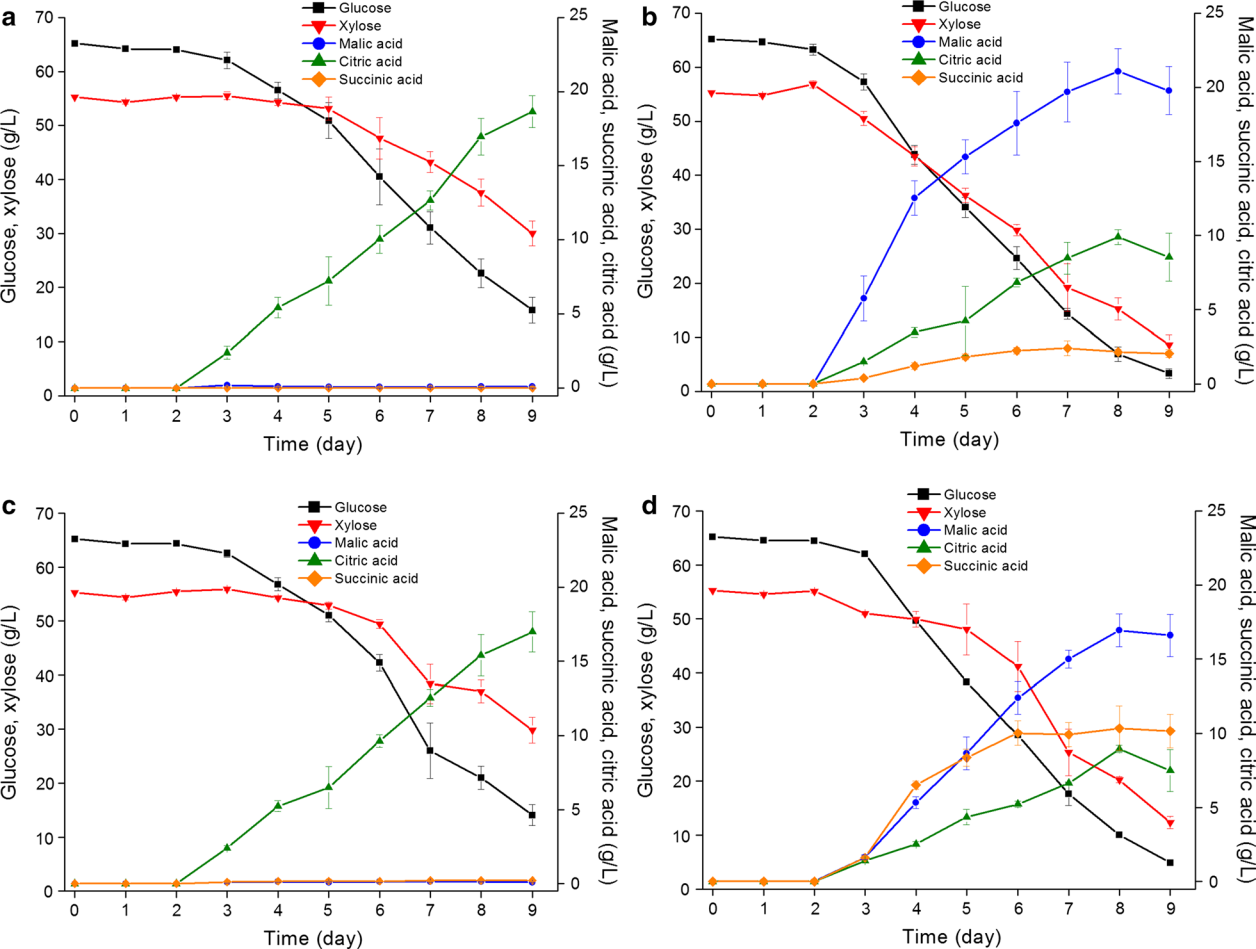
Glucose consumption and production of major organic acids in the wheat straw hydrolysate **a** the parental strain (*∆gox)*; **b** the *dct* strain; **c** the *frd* strain; **d** the *frd*-*dct* strain

The ability of the developed strains to produce C_4_-dicarboxylic acids in the wheat straw hydrolysate buffered with calcium carbonate was investigated. All the tested strains were able to grow in the hydrolysate and utilize glucose and xylose simultaneously for organic acid production (Fig. 5). The concentration of glucose and xylose decreased significantly after day 3, but the sugar consumption varied among the tested strains. After day 3 the *dct* and *frd*-*dct* strains started consuming sugars more rapidly than the parental strain and the *frd* strain, and in total, the *dct* and *frd*-*dct* strains consumed 111 and 105 g/L sugar respectively after 9 days, which was higher than the amounts of sugars consumed by the parental strain (77 g/L) and the *frd* strain (79 g/L). The production of organic acids began in compliance with the sugar consumption in all the tested strains (Fig. 5). The *frd* strain and the parental strain produced higher amounts of citric acid than the *dct* and *frd*-*dct* strains after 9 days. High quantities of malic acid and succinic acid were obtained only in the *dct* and *frd*-*dct* strains. The *dct* strain produced 20 g/L malic acid and 2.1 g/L succinic acid from the hydrolysate, and the *frd*-*dct* strain produced less malic acid (17 g/L) but more succinic acid 10 g/L. Low amount of fumaric acid was also produced by the *dct* and *frd*-*dct* strains (Table 5). For the parental strain and the *frd* strain, low amounts of malic acid and succinic acid but no fumaric acid could be detected in the early phase of cultivation and remained at this level until day 9. Citric acid was produced from day 2 as one of the major organic acids by all the tested strains. The parental strain and *frd* strain produced 19 and 17 g/L citric acid respectively, which were higher than the amounts obtained from the *dct* (8.5 g/L) and *frd*-*dct* strains (7.5 g/L). The fungal biomass of all the tested strains, as well as the organic acid yield, showed the same pattern as obtained in the defined medium. The *dct* and *frd*-*dct* strains had lower biomass yield but much higher yield of C_4_-dicarboxylic acids than the parental strains and the *frd* strain (Table 5).

**Table 5:** Comparison of sugar consumption, production of organic acids and fungal biomass in the wheat straw hydrolysate after 9 days

Strain	Sugar (glucose/xylose) consumption	Concentration (g/L)				Yield (mg/g sugar)				Fungal biomass
		Malic acid	Fumaric acid	Succinic acid	Citric acid	Malic acid	Fumaric acid	Succinic acid	Citric acid	
*∆gox*	77 (51/26)	0.1	n.d	n.d	19	1.4	n.d	n.d	247	515
*dct*	111 (62/49)	20	0.9	2.1	8.5	179	7.9	18	76	342
*frd*	79 (52/27)	0.1	n.d	0.2	17	1.0	n.d	2.8	216	505
*frd*-*dct*	105 (60/45)	17	0.5	10	7.5	157	5.3	96	71.2	345

## Discussion

*Aspergillus carbonarius* is known as an efficient citric acid producing species. It has shown a potential to be a cell factory for production of various organic acids, but the main barrier for C_4_-dicarboxylic acid production is that the intracellular carbon flux primarily flows towards citric acid rather than other acids such as C_4_-dicarboxylic acids when genetic modifications targeting to primary metabolic pathways are introduced.

In this study, a C_4_-dicarboxylate transporter in *A. carbonarius* was identified and overexpressed in order to facilitate the export of C_4_-dicarboxylic acids (malic acid, fumaric acid and succinic acid). Fundamental studies on C_4_-dicarboxylate transporters in microorganisms have focused mainly on bacteria and yeasts e.g. *E. coli* and *S. pombe* [27–29]. The known C_4_-dicarboxylate transporters are normally responsible for transferring several C_4_-dicarboxylic acids rather than a specific one. For instance, the C_4_-dicarboxylate transporters from *E. coli* and *Pseudomonas aeruginosa* can transport malic acid, fumaric acid, oxaloacetic acid, and succinic acid [30, 31]. Although a number of fungal strains are naturally capable of producing high amounts of C_4_-dicarboxylic acids, e.g. *Aspergillus flavus* and *Rhizopus oryzae* [32, 33], there are still very limited amount of information regarding their C_4_-dicarboxylate transporters. The identified C_4_-dicarboxylate transporter from *A. carbonarius* belongs to the same dicarboxylate transporter family as the two reference transporters, C4T318 and MAE1. In *S. pombe*, MAE1 functions as a malate permease transporting C_4_-dicarboxylates via proton symport [27]. Expression of MAE1 in *S. cerevisiae* could increase both uptake and export of C_4_-dicarboxylates depending on the growth conditions [21, 27]. Based on the sequence similarity between MAE1 and the C_4_-dicarboxylate transporter of *A. carbonarius,* the transport of C_4_-dicarboxylates may be achieved via the same mechanism in *A. carbonarius*, and the function of C_4_-dicarboxylate transporter, when it is naturally expressed at a low level, is probably to mediate the uptake of C_4_-dicarboxylates as alternative carbon sources. The recent successes in metabolic engineering for malic acid production have indicated an essential role of C_4_-dicarboxylate transporters for production of C_4_-dicarboxylic acids also in filamentous fungi. Overexpression of a dicarboxylate transporter, C4T318, significantly increased production of malic acid in combination with other genetic modifications in *Aspergillus oryzae* [9]. *A. carbonarius*, compared with *A. oryzae*, is unable to naturally excrete any of the C_4_-dicarboxylic acids. Previous efforts on increasing the carbon flux towards the rTCA branch in *A. carbonarius* led to enhanced production of citric acid but only had a limited impact on malic acid production [16]. This result implies that there exist some limiting steps for C_4_-dicarboxylic acid production in *A. carbonarius*. When the identified putative C_4_-dicarboxylate transporter was overexpressed in the *dct* strain, malic acid production increased dramatically and the titer of malic acid reached 32 g/L in a defined medium after 9 days (Fig. 4b). This indicates that the carbon flux has been partially shunt into malic acid production in the *dct* strain after a more efficient export of malic acid was achieved through overexpression of the C_4_-dicarboxylate transporter. Moreover, the *dct* strain is also able to produce fumaric acid and succinic acid compared with the parental strain, which indicates that the overexpressed C_4_-dicarboxylate transporter, as shown from other known C_4_-dicarboxylate transporters, can transport different C_4_-dicarboxylic acids instead. Accordingly, the *dct* strain produced lower amounts of citric acid (5.3 g/L) than the parental strain (14 g/L), which implies that export of C_4_-dicarboxylate reduced the carbon flux towards biosynthesis of citrate in *A. carbonarius* in buffered conditions. Although there is not yet any study on metabolic flux in pathways related to citric acid production in *A. carbonarius*, the correlation between intracellular concentration of C_4_-dicarboxylates and citric acid production has been illustrated in a well-known citric acid producing strain of *A. niger*, where the increased concentration of cytosolic C_4_-dicarboxylates triggers the citric acid production in the early phase and leads to enhanced citric acid production probably via anti-port of C_4_-dicarboxylates and citrate across mitochondrial membrane [20]. Therefore, the export of C_4_-dicarboxylic acids from the cytosol can theoretically reduce the amounts of C_4_-dicarboxylic acids that are transported into mitochondria for biosynthesis of citric acid and in turn decrease citric acid production in *A. carbonarius*. In addition, the expression of the C_4_-dicarboxylate transporter also influenced the sugar consumption and biomass yield. Compared with the parental strain, the sugar consumption rate increased in the *dct* overexpressing strains after the production of malic acid and succinic acid began at day 3. It seems that the export of C_4_-dicarboxylic acids creates the extra outlet of intracellular carbon flux, which improves the sugar utilization in the *dct* and *frd*-*dct* strains. On the other hand, the fungal biomass decreased in the *dct* and *frd*-*dct* strains compared with the parental strain. This indicates that a re-programming of the carbon metabolism might cause a slow-down of the biomass growth in the derived strains due to the overexpression of the *dct* gene.

While expression of the fumarate reductase from *Trypanosoma brucei* in the natural malic acid producer *Aspergillus saccharolyticus* significantly increased production of succinic acid [22], expression of the fumarate reductase in *A. carbonarius* did not change the production pattern. However, when expressing the fumarate reductase in combination with the C_4_-carboxylate transporter the production of succinic acid was significantly increased. This again indicates the essential role of an efficient succinate export system for enhanced succinic acid production by *A. carbonarius*. As the *dct* strain showed an elevated production of all three C_4_-dicarboxylic acids, it was assumed that the intracellular fumarate can be used as substrate for fumarate reductase to produce succinate in the cytosol and that the overexpressed C_4_-dicarboxylate transporter facilitates the export of succinate across the plasma membrane. As expected, the succinic acid production in the *frd*-*dct* strain increased over twofolds compared with the *dct* strain. This demonstrates the feasibility of improving succinic acid production in *A. carbonarius* by converting fumarate to succinate in the cytosolic reductive pathway.

Currently, the industries for bio-based production of C_4_-dicarboxylic acids are seeking feasible solutions to lower the production cost [10]. Carbohydrates existing in lignocellulosic biomass are considered as cheap alternative substrates for organic acid production [6]. *A. carbonarius* has been reported for its efficient co-utilization of glucose and xylose during the cultivation and its ability to produce different types of organic acids from the hydrolysate such as citric acid and gluconic acid [15]. In this study, we have further demonstrated its ability to produce C_4_-dicarboxylic acids from lignocellulosic biomass with the developed strains. In the hydrolysate, the sugar consumption by all the strains began later than that observed in the defined medium. The inhibitory effects on spore germination and fungal growth in the hydrolysate which delayed sugar consumption in the first two days have been reported in our previous study [15]. However, inoculating with fungal mycelia from pre-culture in this study did not improve the inhibitor tolerance or accelerate the sugar consumption in the early phase of cultivation. Due to the lagged sugar consumption, the organic acid production was also delayed in the hydrolysate. The patterns of organic acids produced by all the tested strains remained the same as that obtained in the defined medium, but the yields of organic acids were lower in the hydrolysate. Fungal organic acid production is significantly affected by a number of factors in the medium, including cultivation pH, carbon sources, nitrogen and metal ions [33–36]. Although the cultivation pH in the defined medium and the hydrolysate was maintained steadily at 6.5 by adding the calcium carbonate, the concentration of nutrients in the hydrolysate was not kept at the same level as in the defined medium due to the complex composition of the wheat straw hydrolysate. This may result in the different yields of C_4_-dicarboxylic acids between these two types of media. For future perspective, the nutrients supplement needs to be optimized based on compositional analysis of hydrolysate to improve C_4_-dicarboxylic acid production.

## Conclusions

This study shows that the key to change the citric acid production of a non-natural C_4_-dicarboxylic acid producing strain into production of C_4_-dicarboxylic acids is the C_4_-dicarboxylate transporter and that the C_4_-dicarboxylic acid production can be further increased via metabolic engineering. Finally, it reveals the potential of *A. carbonarius* to utilize lignocellulosic biomass as substrate for C_4_-dicarboxylic acid production.
